# Histone sumoylation and chromatin dynamics

**DOI:** 10.1093/nar/gkab280

**Published:** 2021-04-22

**Authors:** Hong-Yeoul Ryu, Mark Hochstrasser

**Affiliations:** School of Life Sciences, BK21 FOUR KNU Creative BioResearch Group, College of National Sciences, Kyungpook National University, Daegu 41566, Republic of Korea; Department of Molecular Biophysics and Biochemistry, Yale University, New Haven, CT 06520, USA

## Abstract

Chromatin structure and gene expression are dynamically controlled by post-translational modifications (PTMs) on histone proteins, including ubiquitylation, methylation, acetylation and small ubiquitin-like modifier (SUMO) conjugation. It was initially thought that histone sumoylation exclusively suppressed gene transcription, but recent advances in proteomics and genomics have uncovered its diverse functions in cotranscriptional processes, including chromatin remodeling, transcript elongation, and blocking cryptic initiation. Histone sumoylation is integral to complex signaling codes that prime additional histone PTMs as well as modifications of the RNA polymerase II carboxy-terminal domain (RNAPII-CTD) during transcription. In addition, sumoylation of histone variants is critical for the DNA double-strand break (DSB) response and for chromosome segregation during mitosis. This review describes recent findings on histone sumoylation and its coordination with other histone and RNAPII-CTD modifications in the regulation of chromatin dynamics.

## INTRODUCTION

The small ubiquitin-like modifier (SUMO) is an evolutionarily conserved protein expressed in all eukaryotes (1). Humans express five SUMO paralogs, SUMO-1, -2, -3, -4 and -5, while the budding yeast *Saccharomyces cerevisiae* expresses a single SUMO ortholog, Smt3, that shares 48% identity and 75% similarity with human SUMO-1 (2,3). SUMO proteins modulate the functions of targeted proteins through their dynamic attachment and detachment. SUMO-1, -2, -3 and -5 (and yeast Smt3) are initially translated as C-terminally extended precursors, and the C-terminal tail is cleaved by SUMO-specific proteases to yield mature proteins ending in a pair of glycine residues; the C-terminal α-carboxylate is the site of covalent attachment to other proteins, termed sumoylation (4). In contrast to the other SUMO paralogs, the conjugation capacity of SUMO-4 is unclear because the C-terminal tail apparently cannot be processed *in vivo* (5).

Mature SUMO proteins are covalently attached to lysine (K) side chains of substrate proteins through the activities of an enzyme cascade similar to that in the ubiquitylation pathway (6). Briefly, the SUMO C-terminus is first activated by the heterodimeric SAE1/SAE2 (Aos1/Uba2 in *S. cerevisiae*) SUMO-activating enzyme (E1) and is then transferred to a cysteine in the Ubc9 SUMO-conjugating enzyme (E2). Subsequently, SUMO ligases (E3s) promote the transfer of SUMO from E2 to lysine residue(s) on target proteins. SUMO can also be assembled into polymers on substrates; in yeast, these are specifically disassembled by the Ulp2 SUMO protease. SUMO ligation alters the interactions of substrates with their binding partners; the latter proteins often have one or more SUMO‐interaction motifs (SIMs) that enhance recognition of SUMO-conjugated proteins (7). SIM-containing proteins have emerged as key ‘readers’ of protein sumoylation.

This post-translational modification (PTM) is highly dynamic as it is readily reversed by SUMO-specific proteases. Humans have nine known SUMO-specific proteases, while *S. cerevisiae* expresses two, Ulp1 and Ulp2 (8). Sumoylation of proteins is a critical regulator of many diverse cellular processes, including transcription, DNA replication, cell-cycle progression, mitochondrial dynamics, ribosome biogenesis, DNA repair, apoptosis and stress responses (9,10).

Chromatin structure is centered on nucleosomes, dynamically regulated multiprotein complexes that act as scaffolds for genomic DNA. Each nucleosome is composed of 145 to 147 bp of DNA wrapped around an octamer of histone proteins (two copies each of histones H2A, H2B, H3 and H4) plus a linker histone (H1) involved in higher-order chromatin compaction (11). These histones are subject to multiple PTMs, including sumoylation.

Primary sumoylation of human histone H4 as well as weak sumoylation signals from H2A, H2B and H3 were first observed in 2003 (12), and subsequent studies identified sumoylation at K12 of H4 (13) and K18 of H3 (14). Sumoylation of histone variant H2A.X (15) and H1 (16) were also reported in human cells (Table 1). In *S. cerevisiae*, SUMO can be conjugated to all four core histones (17), as well as the H2A variant H2A.Z (17,18) and H3 variant Cse4 (19). Known SUMO attachment sites of H2B are K6, K7, K16 and K17, while those of H4 are K5, K8, K12, K16 and K20 (17), although there are other potential sites.

**Table 1.** Histone sumoylation sites and functions

Organism	Histone	^a^Discovery	Sites	Function	Refs.
*H. sapiens*	H2A	2003		Transcriptional repression or chromatin compaction	(12,15,16)
	H2B	2003			(12,16)
	H3	2003	K18		(12,14–16)
	H4	2003	K12		(12,13,15,16,28,29)
	H2A.X	2013	K5, K9, K13, K15, K118, K119, K127, K133, K134		(15)
	H1	2009			(16)
*S. cerevisiae*	H2A	2006	^b^K126	Transcriptional repression/activation, inhibition of cryptic initiation	(17)
	H2B	2006	K6, K7, K16, K17		(17,31,40,45)
	H3	2006			(17)
	H4	2006	K5, K8, K12, K16, K20		(17,40,45)
	H2A.Z	2006	K126, K133	^c^DSB repair	(17,18)
	Cse4	2016	K65, K215, K216	Cse4 incorporation or proteolysis	(19,77,78)

^a^The first detected year of histone sumoylation.

^b^H2A sumoylation level was not changed in an arginine substitution mutant of this site.

^c^DSB: double-stranded break.

Among the myriad known histone PTMs, histone sumoylation was discovered relatively recently, so less is known about its effects on chromatin organization and gene expression compared to ubiquitylation, methylation, and acetylation. Although first reported in 2003 (12), most studies on histone sumoylation have appeared within the past five years. Recent investigations utilizing biochemical and genome-wide analyses have contributed much toward our understanding of the patterns and associated functions of this modification. Further, new functions of histone sumoylation continue to be uncovered, and intriguing examples of epigenetic regulation have recently been revealed. The present review provides an overview of newly discovered functions for histone sumoylation, including the dynamic regulation of eukaryotic chromatin structure and transcription.

## HISTONE SUMOYLATION AND TRANSCRIPTION

### Histone sumoylation in transcriptional repression

Many SUMO target proteins are transcriptional co-activators or co-repressors (20–23), suggesting that sumoylation may have both positive and negative effects on the expression levels of diverse gene types, including constitutively expressed and inducible genes (23). The first report of histone sumoylation in human cells by Shiio and Eisenman suggested a negative effect on transcription because an engineered SUMO−H4 translational fusion associated with the transcriptional repressors histone deacetylase 1 (HDAC1) and heterochromatin protein HP1 in cells (12). The authors observed SUMO-1 attachment to acetylated H4 and enhancement of this sumoylation reaction by co-expression of the histone acetyltransferase (HAT) p300, suggesting that histone acetylation may facilitate subsequent SUMO conjugation to H4 (12); these results provided an early hint of the complex interplay of histone sumoylation with other histone PTMs. In the case of non-histone substrates, it has also been reported that SUMO-modified p300 and CREB-binding protein mediate transcriptional repression by promoting recruitment of HDAC6 (24). Despite these early results, there has been relatively limited study of transcriptional repression mechanisms by histone sumoylation until recently.

**Figure 1.**
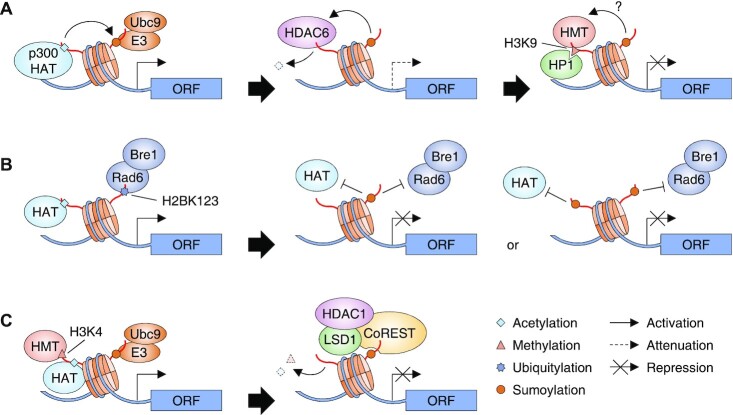
Models for the functions of histone sumoylation in transcriptional repression. (**A**) During switching from transcriptional activation to repression in mammals, p300 HAT-mediated histone acetylation promotes histone sumoylation by activating Ubc9 and SUMO E3 ligase. Sumoylated histones then recruits both HDAC6, which attenuates transcription, and HP1, which contributes to chromatin compaction. However, it is still unclear whether histone sumoylation stimulates H3K9 methylation, a marker for HP1 binding. (**B**) Histone sumoylation in yeast potentially interferes with histone acetylation by HATs or H2BK123 ubiquitylation by Rad6 and Bre1, thereby inhibiting transcription. (**C**) LSD1−CoREST−HDAC1 complex is associated with sumoylated histone through the SUMO-interacting motif (SIM) in the CoREST subunit, allowing LSD1 and HDAC1 to reverse H3K4 methylation and histone acetylation, respectively; both of the latter histone marks normally promote transcription.

Gene expression levels are tightly regulated by co-activators and co-repressors that promote reversible switching between ‘on’ and ‘off’ states. Histone acetylation is a major driver of the transcriptionally active chromatin state, while ensuing sumoylation may provide reciprocal control to limit expression. Indeed, in follow-up studies to those noted above, histone acetylation was found to stimulate Ubc9-mediated histone sumoylation, and conversely, p300-mediated gene activation is repressed by histone-SUMO modification and subsequent HDAC6 recruitment in human cells (Figure 1A) (24). Such histone sumoylation also leads to condensed chromatin and gene silencing by facilitating the recruitment of HDAC1 and HP1. However, it has yet to be determined whether histone sumoylation affects H3K9 methylation, a marker of HP1-mediated gene repression (25).

The sophisticated molecular genetic tools available for studying the yeast *S. cerevisiae* were employed not long after these first mammalian studies, allowing important advances in our understanding of histone sumoylation. To circumvent the lack of sumoylated histone-specific antibodies, Berger and colleagues evaluated yeast histone H2B sumoylation levels by a two-step chromatin double immunoprecipitation (ChDIP) protocol in cells expressing H2B tagged with a Flag epitope and SUMO (Smt3) tagged with an HA epitope; anti-Flag beads were used to isolate Flag-H2B in the first step and anti-HA beads against HA-SUMO were utilized in the second step (17,26). Sumoylated H2B was observed at many genomic locations, including the galactose-inducible *GAL1* gene, with slightly stronger signals at subtelomeric regions.

Alanine substitutions at K6, K7, K16 and K17 of H2B (H2B-4KA) strongly reduced its conjugation to SUMO and led to modest increases in expression of several tested genes, including *GAL1*, under non-inducing conditions, in *S. cerevisiae* (17). Conversely, direct fusion of SUMO to H2B (or H3) strongly reduced expression of *GAL1* under inducing conditions. Increased H2B or H4 sumoylation correlated with decreased histone acetylation (H2BK16Ac) under *GAL1*-inducing conditions as well as in mutants with a substitution at the ubiquitylation site H2BK123. Moreover, H3 acetylation was enhanced in a *ubc9ts* strain (a temperature-sensitive E2 mutant) and in cells lacking the Siz1 and Siz2 SUMO E3 ligases. Collectively, these findings suggest that histone sumoylation may be involved in transcriptional repression via inhibition of, or competition with, histone epigenetic marks for gene activation such as ubiquitylation and acetylation (Figure 1B).

### Histone sumoylation and chromatin structure

Two critical issues that remained unresolved in these early studies were (i) whether histone sumoylation results exclusively in transcriptional repression or if transcriptional activation is also possible, and (ii) whether individual genes can be regulated by specific patterns of histone sumoylation. An early proteomics analysis of HeLa cells identified the K12 residue of H4 as a site of SUMO-3 conjugation (13). Chatterjee and colleagues employed a disulfide-directed protein modification strategy, which uses disulfide chemistry to crosslink two peptides (27), and generated a human histone H4K12C protein crosslinked to the C-terminus of SUMO (28). Nucleosomes assembled *in vitro* with this SUMO-modified version of H4 were less stable and were unable to form dinucleosomes *in vitro*, similar to nucleosomes bearing K16-acetylated H4, a known marker of open chromatin. These findings suggest that sumoylation at H4K12 also inhibits chromatin compaction by inhibiting inter-nucleosomal interactions.

These results would appear to be at odds with the earlier reports (12,17), which had implied a role for histone sumoylation in forming closed chromatin structures. Subsequent experiments, however, provided evidence that could reconcile these data. In particular, these analyses showed that SUMO-3-conjugated H4 stimulates lysine-specific demethylase 1 (LSD1)-mediated removal of H3K4 methylation *in vitro*; H3K4 methylation is a sign of active chromatin (29). To effect transcriptional repression, LSD1 associates with CoRepressor for Element 1 Silencing Transcription factor (CoREST) and HDAC1 (30). Importantly, a SIM in CoREST is required for H3K4 demethylation by LSD1, but this demethylation activity is not propagated into adjacent nucleosomes. Chatterjee and colleagues suggested that transient histone sumoylation may provide a binding platform for CoREST, LSD1 and HDAC1, allowing spatially restricted gene repression by clearance of local PTMs that would otherwise promote transcription (Figure 1C). Their model is consistent with the low level of histone sumoylation in cells (17) because subsequent elimination of SUMO modifications is required to suppress its negative effects on chromatin compaction and to establish silenced heterochromatin (29).

**Figure 2.**
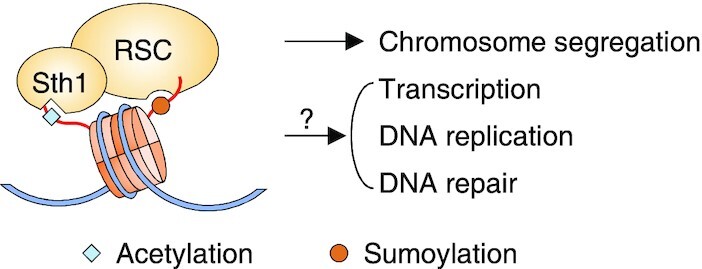
Histone sumoylation promotes chromatin binding of RSC. The Sth1 subunit of RSC recognize H3K14 acetylation, and an unknown RSC component recognizes sumoylated histones. This dual recognition has been implicated in chromosome segregation, but its function in other RSC-controlled processes has not yet been determined.

A very recent study identified another possible role for histone sumoylation in the regulation of chromatin structure in *S. cerevisiae* (31). The Remodeling the Structure of Chromatin (RSC) complex is a member of the ATP-dependent nucleosome remodeler family (32) that alters the position, occupancy, and composition of nucleosomes in chromatin; RSC activity regulates transcription (33–35), DNA replication (36), chromosome segregation (37), and DNA repair (38,39). DNA footprint analysis of nucleosome-associated Sth1, the catalytic subunit of the RSC complex, revealed that H3K14 acetylation facilitates nucleosome binding of RSC by association with the C-terminal bromodomain of Sth1 (31). Furthermore, yeast mutants with substitutions of the SUMO sites in H2B or loss of the SUMO ligases Siz1 and Siz2 displayed impaired association of RSC with nucleosomes *in vivo*; conversely, nucleosomes containing SUMO-fused H2B showed greater *in vitro* binding to RSC than did unmodified nucleosomes. This suggests that SUMO-histone conjugation promotes binding by RSC; however, the relevance of this mechanism (Figure 2) to RSC-controlled cellular processes such as transcription or replication still needs to be demonstrated.

Taken together, these findings suggest that histone sumoylation can suppress the formation of certain higher-order chromatin structures and acts as a crucial signal for recruitment of factors involved in gene activation as well as repression.

### Histone sumoylation is integral to multiple transcriptional regulatory cascades

An early study reported only mono-sumoylated histones in WT and *ulp2Δ* yeast cells (17). However, recent work conducted under experimental conditions that preserve most sumoylated species has also identified polymeric SUMO chains on histones H2B and H4 (40). SUMO chains can act as complex signaling codes to guide subsequent protein activity, including addition or removal of other PTMs (41,42). For yeast histones, poly-SUMO chains are efficiently disassembled by the Ulp2 SUMO protease (40), so they are highly dynamic. Genome-wide localization studies have identified enrichment of SUMO-conjugated H2B and Ulp2 at the loci of constitutively transcribed genes, activated inducible genes, and genes encoding ribosomal proteins (40,43–46).

**Figure 3.**
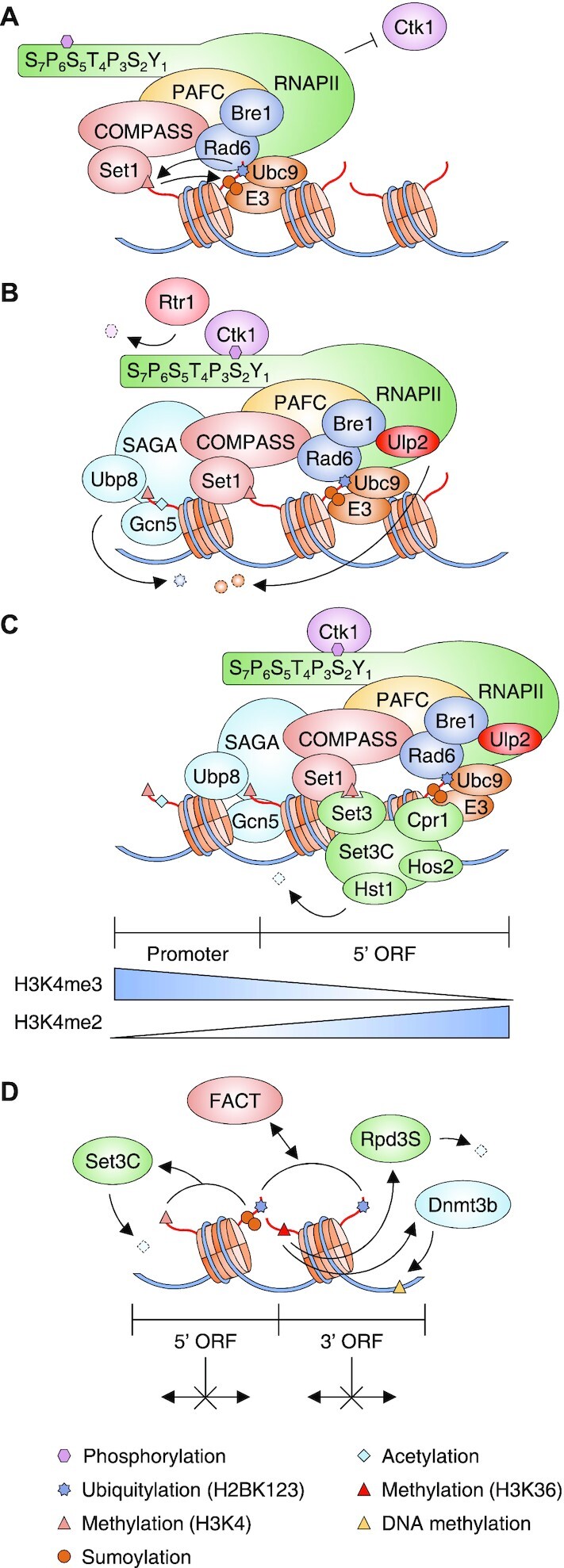
Model of histone sumoylation in the prevention of cryptic initiation. (A–C) Illustrations show the relevant components, but not the precise physical association or order of events. Triangles at the bottom indicate gradients of H3K4me3 and H3K4me2 modifications over the promoter and 5′ regions of the open reading frame (ORF). (**A**) At the early stage of transcription, the CTD S5 phosphorylated forms of RNAPII and PAFC are required for H2BK123 ubiquitylation by Rad6 and Bre1. H2B ubiquitylation drives two sequential modifications, COMPASS/Set1-mediated H3K4 methylation and histone poly-sumoylation by Ubc9 and a SUMO E3. Both H2B ubiquitylation and histone sumoylation inhibit Ctk1 (the major S2 kinase) association with the RNAPII transcription machinery. (**B**) Ubiquitin removal from histones by SAGA component Ubp8 and polySUMO disassembly by Ulp2 together facilitate Ctk1 recruitment and CTD S2 phosphorylation for subsequent transcription elongation, while Rtr1 dephosphorylates S5 in the CTD. The Gcn5 HAT, another SAGA subunit, mediates histone acetylation during transcription elongation. (**C**) In the transcription elongation step, repeated rounds of H2B ubiquitylation and histone sumoylation and their reversal occur while an H3K4 methylation gradient is gradually established. Recognition of H3K4me2 by Set3 and sumoylated histones by Cpr1, both subunits of the SET3C deacetylase, is required for recruitment of SET3C to the 5′ regions of ORFs. Hst1 and Hos2, the catalytic subunits of SET3C, block accumulation of hyperacetylated histones in these ORF regions. (**D**) Inhibition of spurious transcription initiation by cotranscriptional histone modifications. H2B ubiquitylation functions cooperatively with the FACT complex to suppress cryptic transcription of genes. H3K4 methylation and histone sumoylation facilitate histone deacetylation by SET3C in 5′ ORF regions, and H3K36 methylation promotes histone deacetylation by Rpd3S in 3′ ORF regions. In mammals, Dnmt3b-mediated DNA methylation restricts the generation of cryptic transcripts in a H3K36 methylation-dependent manner.

While histone modifications were once considered independent PTMs, it is now becoming clear that modifications at different sites can exhibit interdependence under specific conditions (context-dependent crosstalk), which has important implications for the control of chromatin dynamics (47). For example, yeast histone sumoylation may interfere with or counteract H2B mono-ubiquitylation (17), while other evidence suggests that H2B and H4 sumoylation may require H2B ubiquitylation mediated by Rad6 (E2) and Bre1 (E3) (40). The Ubp8 deubiquitylase-mediated removal of ubiquitin from H2B is also required for nucleosome binding to Ctk1 kinase (48). Ctk1 in turn phosphorylates serine-2 (S2) within the C-terminal domain (CTD) Y_1_S_2_P_3_T_4_S_5_P_6_S_7_ heptad repeat region of Rpo21/Rpb1, the largest RNA polymerase II (RNAPII) subunit; this is known to promote transcriptional elongation and couple it to mRNA 3′ end processing (49,50). The association between the nucleosome and Ctk1 is blocked by both H2B ubiquitylation and sumoylation, and Ulp2-dependent desumoylation of histone facilitates later transcriptional elongation steps by promoting Ctk1 recruitment (40). Removal of the phosphate on the CTD S5 residue by the Rtr1 phosphatase is also required for transcriptional elongation (51) (Figure 3A and B). These data describing sequential histone modification changes provided the first suggestion that histone sumoylation may also be involved in transcriptional activation.

The prototypical example of histone crosstalk is the ‘histone trans-tail pathway’ involving H2B ubiquitylation-dependent K4 and K79 methylation of H3 (52,53). Intriguingly, H2B ubiquitylation-mediated H3K4 dimethylation (me2), but not trimethylation (me3), is also required for subsequent sumoylation at H2B and H4 during transcription (45). Methylation of H3K4 exhibits an intrinsic gradient pattern, with me3 more frequent near the promoter, me2 in the 5′ region of the open reading frame (ORF), and monomethylation (me1) in more gene-distal regions (54,55). The degree of H3K4 methylation is determined by the amount of time the Set1 methyltransferase is tethered near the nucleosome during multiple rounds of transcription (56). Notably, H3K4me2 has a function distinct from that of H3K4me3 in transcription by providing a binding site for the PHD finger within Set3, a subunit of the Set3 complex (SET3C); SET3C is a histone deacetylase that includes two active HDAC subunits, Hos2 and Hst1 (57,58). SET3C-mediated histone deacetylation in the 5′ ORF region contributes to the suppression of cryptic initiation of both sense and antisense RNA transcription from within the ORF (58,59).

In addition to SET3C binding to H3K4me2, yeast SET3C also preferentially associates both *in vivo* and *in vitro* with SUMO-modified histones via a SIM in Cpr1, another subunit of SET3C (45). Importantly, the changes in noncoding RNA (ncRNA) expression exhibited by cells lacking Set3 strongly overlapped with those in cells exclusively expressing the H2B-4KA mutant (which strongly reduces its sumoylation), implying a strong association between SET3C function and histone sumoylation (45). Notably, the H2B-4KA mutations lead to dramatic decreases in Set3 and Cpr1 occupancy at target genes and increases in spurious transcription of sense ncRNAs initiated from cryptic internal promoters.

**Figure 4.**
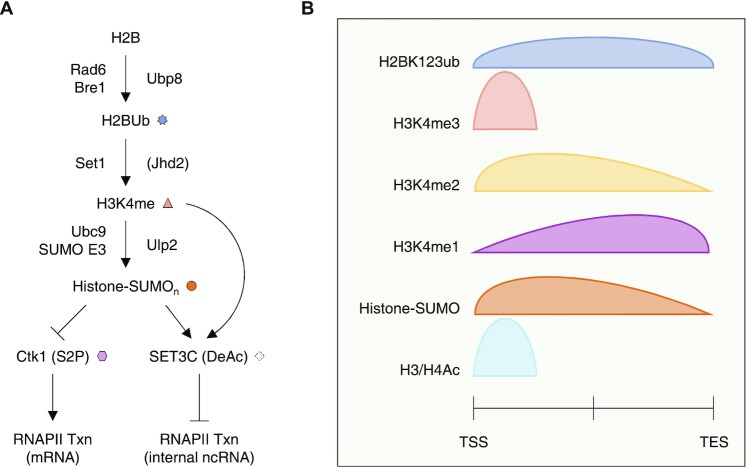
Genome-wide distribution pattern of histone modifications that crosstalk with histone sumoylation in active genes. (**A**) Schematic diagram depicting the distinct roles of histone sumoylation in transcription and its crosstalk with other histone modifications. (**B**) The genomic localization of histone modifications is mapped on a generalized gene aligned from transcription start site (TSS) to transcription end site (TES). The curves represent their distribution patterns determined by genome-wide analyses in yeast.

Taken together, these results indicate that sequential histone modifications—H2B ubiquitylation, H3K4 methylation, histone sumoylation and histone deacetylation—function in a complex crosstalk pathway to prevent inappropriate internal transcription within gene coding sequences (Figures 3A–C and 4). This mechanism is distinct from other histone modification-dependent mechanisms that also contribute to the suppression of spurious transcription initiation, namely, interdependent regulation of nucleosome reassembly by H2B ubiquitylation and the FACT complex and H3K36 methylation-mediated association of the Rpd3S HDAC complex or Dnmt3b DNA methyltransferase (Figure 3D) (60–63). In summary, the role of histone sumoylation in transcription cannot be simply defined as ‘positive’ or 'negative’. Instead, crosstalk with other histone modifications both regulates transcriptional elongation and maintains transcriptional fidelity by an elaborate regulation of transcription steps (Figure 4A).

### Genome-wide maps of histone sumoylation and other histone modifications

The genome-wide localization of histone modifications associated with histone sumoylation has been examined to uncover their potential roles in yeast transcription (Figure 4A and B). H2B ubiquitylation appears at an early stage among the dynamic changes in histone PTMs and chromatin during transcription; it is preferentially enriched across transcribed regions and correlates positively with transcriptional gene activity (64–66) (Figure 4B). In turn, H3K4 methylation, which requires H2BK123 ubiquitylation, is distributed in distinct gradients relative to the transcribed DNA sequence that depend on the extent of H3K4 methylation (56,67). The genome-wide localization pattern of SUMO-modified histones correlates closely with the H3K4me2 profile on actively transcribed genes (45), supporting the idea that these two modifications contribute to the same or a similar step of chromatin-mediated transcription. Finally, a strong peak of acetylated histones H3 and H4 is detected upstream of the histone-SUMO and H3K4me2-enriched regions in active genes (55,68), consistent with histone sumoylation and H3K4me2 working in concert to recruit SET3C for histone deacetylation.

## HISTONE SUMOYLATION IN DSB REPAIR

Genomic DNA suffers double-strand breaks (DSBs) throughout an organism's life due to genotoxic agents (such as γ-irradiation) or physiological processes such as meiosis (69). Therefore, DSB repair is an essential step for cell survival and the maintenance of genome integrity. Repair is mediated by either error-prone nonhomologous end-joining (NHEJ) or homologous recombination (HR), which utilizes sequences homologous to the broken DNA to guide accurate repair (70,71). In the HR repair pathway, yeast histone variant H2A.Z is required to create an open chromatin structure (72,73). Upon induction of a persistent DSB without available DNA homology for repair, H2A.Z is rapidly loaded near the break site to drive the relocation of the unrepaired chromosomal ends toward the nuclear envelope and then is slowly removed (18). While only a low level of SUMO-modified H2A.Z was originally reported in yeast cells during DSB repair (17), a subsequent investigation found that H2A.Z incorporated into nucleosomes at a persistent DSB site was sumoylated and that this sumoylation was required for DSB tethering to the nuclear periphery (18). It has not been determined how SUMO modification of H2A.Z affects DSB relocation, but a key recombination factor in the DSB response, Rad52, is a known SUMO substrate (74,75).

## HISTONE SUMOYLATION AT THE CENTROMERE

**Figure 5.**
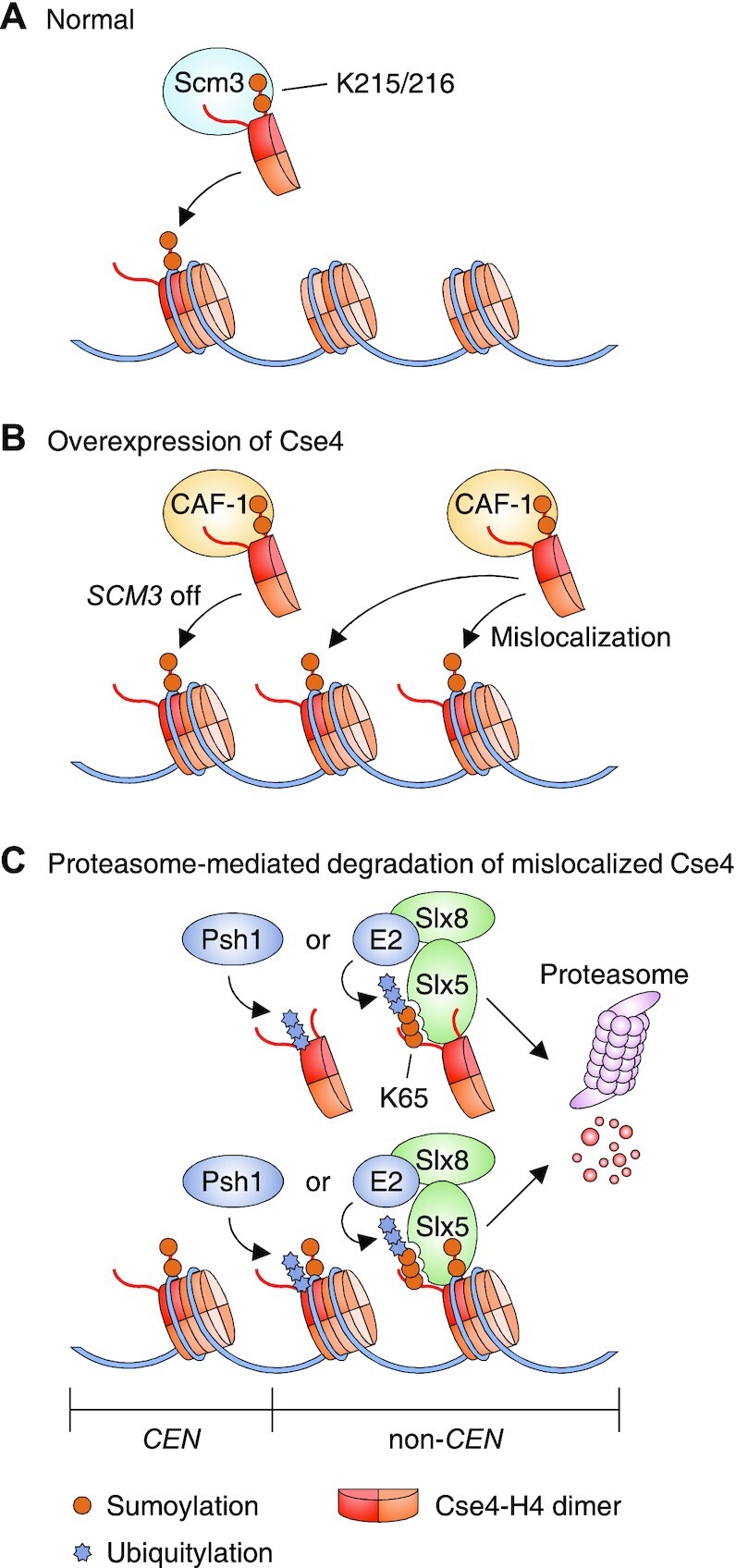
Sumoylation of Cse4 mediates its appropriate localization. (A and B) Cse4-K215/216 sumoylation triggers Scm3-dependent incorporation of Cse4−H4 dimers into the *CEN* regions of chromosomes in normal cells (**A**), while CAF-1 also interacts with K215/216-sumoylated Cse4 and promotes deposition of overexpressed Cse4−H4 dimers into non-*CEN* regions (and *CEN* domains when Scm3 levels are low) (**B**). The SIMs of Scm3 and CAF-1 are not yet determined. (**C**) Sumoylation of K65 in Cse4 limits its levels or prevents its mislocalization in a manner dependent on Slx5/Slx8-mediated ubiquitylation and proteasome-mediated proteolysis. The Psh1 ubiquitin ligase independently facilitates proteasomal degradation of mislocalized Cse4.

Recent studies have demonstrated a specific role for sumoylation of the *S. cerevisiae* histone H3 variant Cse4 (human CENP-A) in mitosis. The incorporation of Cse4 into centromeric nucleosomes is required for normal kinetochore assembly and chromosome stability, and thus ultimately for faithful chromosome segregation (76). The Cse4 protein is a substrate for Siz1 and Siz2 SUMO ligases *in vitro* and *in vivo* (19), and subsequent biochemical studies revealed the major SUMO-Cse4 conjugation sites (77,78). Sumoylation at C-terminal K215/216 sites of Cse4 facilitates its association with the Cse4-specific histone chaperone Scm3 (78), promoting Cse4 deposition at centromeres (Figure 5A). The chromatin assembly factor-1 (CAF-1) complex also interacts with K215/216-sumoylated Cse4 and drives overexpressed Cse4 into incorrect, noncentromeric sites (78) (Figure 5B). However, CAF-1 can also trigger the deposition of Cse4 into the centromeric region when *SCM3* gene expression is reduced (78).

Interestingly, sumoylation of Cse4 at a different site, K65, prevents the aberrant spread of Cse4 into euchromatin by providing a signal for ubiquitylation by the Slx5/Slx8 SUMO-targeted ubiquitin ligase (STUbL), which leads to Cse4 degradation by the proteasome. An alternative E3 ubiquitin ligase, Psh1, can also mark mislocalized or excess Cse4 for proteasomal degradation by a SUMO-independent mechanism (19,77) (Figure 5C). A reduction in histone H4 dosage prevents the ectopic localization of overexpressed Cse4, which correlates with reduced Cse4 sumoylation (79). The data imply that noncentromeric deposition of excess Cse4 is mediated by Cse4 sumoylation in the context of H4-Cse4 dimers. Overexpression and mislocalization of CENP-A, the human ortholog of Cse4, have been observed in many cancers and lead to aneuploidy, defined as the presence of an abnormal number of chromosome copies (80–88). Therefore, further study of SUMO modification of this H3 variant may provide a better understanding of tumor development and reveal potential new strategies for cancer treatment.

## PERSPECTIVE

Since the first evidence for SUMO conjugation of histone proteins ∼18 years ago (12), multiple investigators have demonstrated potential functions for histone-SUMO conjugates in the epigenetic regulation of gene expression as well as the DNA DSB response and chromosome segregation. However, mechanistic studies have been largely limited to yeast and to mammalian cells *in vitro*. Therefore, many of the mechanisms discussed still need to be extended by *in vivo* studies of mammals, including analyses of the roles of histone sumoylation in tumorigenesis.

There are several hurdles yet to overcome for a more detailed understanding of histone sumoylation in epigenetic gene regulation and other genomic control processes. Of particular note, we do not know how the Ubc9 E2 or SUMO E3 ligases are able to sumoylate histones in a chromosomal site-specific way. Although some evidence indicates such enzymes can be recruited to distinct loci, these proteins do not possess obvious domains for DNA binding or histone modification recognition, such as ubiquitin-binding or methylated lysine-binding domains (43,89). Proteins such as transcriptional activating or silencing factors may also help to localize SUMO pathway enzymes to specific chromatin sites.

Major experimental challenges include preservation and detection of the low-abundance sumoylated forms of histones and unambiguous determination of sumoylation sites (6,17). Unfortunately, overexpression of SUMO proteins by increased SUMO gene dosage, promoter swapping, or environmental changes such as heat shock can also alter the normal levels, intracellular location, or biological activity of SUMO targets as well as the complex crosstalk among protein PTMs (90). It is hoped that advances in quantitative proteomics and the development of site-specific antibodies to SUMO-conjugated histones and other sumoylated chromatin factors will provide powerful and unbiased approaches to identify sumoylated proteins and their modification sites.

Understanding the physiological impact of the complex signaling codes conferred by sumoylation and other PTMs of histones will require identifying their full range and dynamics. Moreover, SUMO proteins are themselves subject to PTMs such as phosphorylation, acetylation, and ubiquitylation (91–95), and also have internal sumoylation sites, resulting in the formation of SUMO chains (14,41). Improved mass spectrometry and peptide sequencing technology will facilitate the identification of complex PTMs, including those of SUMO (96). While the cues provided by these complex codes have not been elucidated in most instances, several studies have provided early clues about how a few of them specify downstream biological events. For instance, the BRCA1-A complex subunit RAP80 recognizes mixed SUMO-ubiquitin chains formed at sites of DNA damage and thus can recruit the BRCA1 DNA repair complex to these sites (97). Combinations of histone sumoylation and other histone modifications or the variously phosphorylated forms of RNAPII-CTD also provide potential signals to guide transcription and other chromatin-dependent processes. To understand these signals, many more single-gene and genome-level analyses will be required.

SUMO modification is essential for myriad cellular processes in all eukaryotes and is implicated in diverse diseases (98). In addition, dysfunctional RNAPII CTD phosphorylation and histone ubiquitylation, methylation, and acetylation, all of which interact with histone sumoylation, are strongly implicated in neurodegenerative diseases and cancer (99–103). Thus, in addition to revealing new insights into the epigenetic mechanisms regulating gene expression, chromatin structure, and genome stability, understanding the functions of histone sumoylation will likely provide new therapeutic strategies and drug targets for disease treatment.

## DATA AVAILABILITY

All data in the referenced studies published by the present authors are available from the authors or have been deposited in public databases.
